# Supplementary material for: Sodium nitrite promotes atherosclerosis via IL-1β: Network toxicology and machine learning insights
Source: PLoS One. 2026 May 5;21(5):e0347642. doi: 10.1371/journal.pone.0347642 (PMC13143078; doi:10.1371/journal.pone.0347642)
Supplement: Attachment — Submitted filename: Response to Reviewers.docx [file pone.0347642.s003.docx]

Journal：PLOS One

Manuscript Number:PONE-D-25-58627

Dear Editor of PLOS One,

Thank you very much for your letter and the reviewers' comments to improve the quality of the manuscript, based on which we have carefully revised the manuscript. The point-by-point responses to the comments are detailed in the following part. We hope these changes can make the manuscript more acceptable. There are two manuscript files, one is "Marked Revision", in which all the modifications are highlighted, and the other (Revised Manuscript) is the revised version ready for publication. If any further clarification and improvement are required, please do not hesitate to let us know. We look forward to hearing from you soon. Additionally, the source code related to this manuscript has been uploaded to GitHub and is publicly accessible at the following link: [https://github.com/fierydoctor9988/SodiumNitrite_Atherosclerosis_Code].

Note that the following color scheme is used in responding to reviewers' comments:

Black:reviewers' comments

**Blue:**our responses

***Blue:our revisions in the manuscript (italic)***

**Reviewer#1**

**Dear Professor,**

**Comment 1: The manuscript mentions that targets related to sodium nitrite were collected from GeneCards, ChEMBL, STITCH, and CTD databases, with atherosclerosis (AS)-related targets sourced from OMIM and GeneCards. However, GeneCards returned thousands of genes based on relevance scores, and the manuscript only states that "duplicate entries were removed" without specifying a clear cutoff value for the relevance scores. Including all genes could potentially lead to false-positive targets, compromising the reliability of the results. Therefore, we kindly request that the authors specify the exact filtering criteria applied to the GeneCards database and provide the rationale for selecting these criteria. Additionally, for the STITCH database, the confidence score settings should be reported.**

R: Thank you for your professional guidance. We have clarified and screened the top 2000 genes among the 6637 AS related genes provided by the GeneCards database, with a minimum correlation score threshold of 1.26, in order to reduce the risk of false positives and ensure the reliability of the results. In addition, we have added parameter settings for the STITCH database: the species is Homo sapiens, and the confidence score is ≥ 0.7. This threshold aims to incorporate interactions with high confidence and more sufficient evidence support, thereby avoiding interference from low confidence predicted items on subsequent results.

**The revised content is as follows:** For GeneCards, the top 2000 genes based on relevance scores were selected. In the case of STITCH, only interactions with a confidence score ≥ 0.7 were considered.

[This modification is located on page 4, lines 90‒92.]

**Comment 2: The finding that approximately 70% of sodium nitrite's targets (34 in total) overlap with disease targets suggests a close association between sodium nitrite and atherosclerosis (AS). However, conversely, the possibility that the initial target collection was overly broad cannot be ruled out. I recommend that the consideration section thoroughly address whether the 24 targets represent direct binding targets of sodium nitrite or indirect effects via downstream signalling mechanisms.**

R: Thank you for your insightful comment. We have clarified in the results that the 24 overlapping genes should be inferred as putative targets from database associations, rather than confirmed direct binding targets of sodium nitrite. At present, bioinformatics databases cannot distinguish whether target genes directly bind to targets or indirectly downstream targets. In addition, we explicitly clarified this point in the discussion to avoid overinterpretation and emphasized the need for mechanical validation in future work to determine the direct and indirect nature of these associations.

**The revised content is as follows:** The 24 overlapping genes were derived from public database annotations and should be considered hypothesis-generating putative sodium nitrite-AS-related targets rather than confirmed direct binding targets, because the overlap may reflect indirect downstream effects, and thus requires future in vitro/in vivo sodium nitrite exposure studies for validation.

[This modification is located on page 27, lines 484‒487.]

**Comment 3: The abstract and main text claim that ‘multi-omics bioinformatics’ was performed. However, the actual analyses conducted were limited to Transcriptomics (GEO datasets). Direct integrated analysis of Metabolomics or Proteomics data was not performed; instead, it remains confined to a literature review. The term ‘multi-omics’ should be revised to something like ‘Transcriptomics integrated with Network Toxicology’, or results from the actual integrated analysis of other omics data should be added. The current phrasing may mislead readers.**

R：Thank you for your insightful suggestion. To ensure objectivity and rigor, we have revised the abstract to more accurately describe our analysis as a combination of transcriptomics and network toxicology. We also clarified in the manuscript that a literature review was conducted on metabolomics and proteomics, but these data were not directly included in the current analysis.

**The revised content is as follows:** To address this gap, we integrated network toxicology, machine learning, transcriptomics, molecular docking, and molecular dynamics simulations. Disease-related targets were first identified from public databases, and four core candidates—IL-1β, IL6, PTK2, and NOS3—were prioritized using machine learning approaches.

[This modification is located on page 1, lines 16.]

**Comment 4: The reviewer raises concerns regarding the machine learning techniques (LASSO and SVM-RFE) used to select key genes, specifically regarding the input data (Feature Matrix) and the context of the samples used. The reviewer notes that while it seems the expression levels from the GEO dataset (GSE28829) were used, this dataset pertains to AS patient samples rather than samples exposed to sodium nitrite. Therefore, the reviewer questions whether the selected genes are biomarkers for AS or toxicity targets for sodium nitrite, and whether the machine learning results alone can definitively prove the causal relationship between sodium nitrite and these genes.The reviewer also requests clarification in the Methods section regarding the training data used for the machine learning models, including the number of samples, group information, etc. Additionally, the reviewer suggests that to substantiate that the selected genes represent “targets of sodium nitrite”, it is important to demonstrate how these genes either match the initial 34 sodium nitrite-related targets or at least have a significant connection to them.**

R: Thank you for your constructive suggestion. We agree on the importance of clearly explaining the input data and the relationship between sodium nitrite and the selected genes. In this study, machine learning was used to analyze transcriptome data based on the GEO dataset GSE28829 (29 samples, including 16 AS samples and 13 control samples), and specifically applied to the expression matrix of 34 sodium nitrite AS related candidate targets. Ultimately, 24 genes were selected to distinguish AS from the control group. We acknowledge that the lack of appropriate datasets for sodium nitrite exposure limits the possibility of multi model validation, therefore the current analysis focuses on AS patient samples. We have revised the methodology section to clarify the data composition and point out this limitation. We emphasize the need for further in vitro and in vivo studies using sodium nitrite exposure models in the future to verify whether these genes are directly regulated by sodium nitrite or reflect indirect pathological effects of AS.

**The revised content is as follows:** The feature matrix consisted of expression values of the 34 overlapping sodium nitrite–AS candidate targets in GSE28829 (n=29; AS=16, control=13).

[This modification is located on page 5, lines 102-103.]

**Comment 5: The reviewer raises a concern regarding the risk of data leakage and overfitting when performing both feature selection and model validation (e.g., using the ROC curve) on a single GEO dataset, especially when the dataset has a limited number of samples.Secure an external validation dataset (e.g., another GEO dataset) to revalidate the diagnostic capability of the four genes, or if obtaining an external dataset is difficult, you should explicitly state whether the training/test set separation was strictly followed during the internal validation process to reduce the risk of overfitting and provide transparency to the readers.**

R: Sincerely thank the reviewer for their insightful comments on the potential risks of data leakage and overfitting during feature selection and validation in a single queue. In response, we conducted external validation using the independent GEO dataset GSE100927 to re evaluate the diagnostic performance of the four key genes identified by the machine learning program. In the external validation queue, IL-1 β exhibited the strongest and most robust diagnostic performance, with an AUC of 0.822 (95% CI: 0.7372-0.9069, DeLong), sensitivity of 70.4%, and specificity of 84.8% at the optimal critical value determined by the Youden index. The remaining genes exhibit relatively low discriminatory abilities. These results have been added to the Results section, and the corresponding ROC curves are shown in the revised Figure 6. In addition, we proposed in the discussion that future in vitro and in vivo studies using sodium nitrite exposure models are necessary for establishing direct causal relationships and verifying the role of target mechanisms.

**The revised content is as follows:** External validation was conducted using GSE100927. ROC curves were generated with the pROC package, and AUCs with 95% confidence intervals were calculated using DeLong’s method. The optimal cutoff was determined by the Youden index, and sensitivity and specificity were reported.

[This modification is located on page 6, lines 132-135]

In comparison, the AUCs of the other three genes in GSE100927 were PTK2 (0.751), IL6 (0.471), and NOS3 (0.618), indicating that IL-1β was the most consistently discriminative marker across datasets(Fig.6A-D).To evaluate the performance of the model, the accuracy, recall, and F1 score of the core objective were also calculated. The accuracy of IL-1β is 1, the recall rate is 0.875, and the F1 score is 0.933, showing excellent performance in distinguishing AS related biomarkers. These results emphasize the robustness of the model and support the diagnostic relevance of IL-1β in atherosclerosis.

Fig 6.External validation of diagnostic performance for the four key genes in GSE100927. (A) IL-1β. (B) IL6. (C) NOS3. (D) PTK2.

[This modification is located on page 12, lines 232-239]

To further reduce the potential risk of overfitting from single-cohort validation, we performed external validation using an independent GSE100927. In this external cohort, IL-1β showed the best-performing and most robust diagnostic discrimination, achieving an AUC of 0.822 (95% CI: 0.7372–0.9069, DeLong). Using the Youden index, the optimal cutoff (7.058918) yielded a sensitivity of 70.4% and a specificity of 84.8%. Notably, GSE100927 represents an AS cohort without sodium nitrite exposure information; therefore, this external validation supports the reproducibility of IL-1β as an AS-related diagnostic marker rather than providing direct evidence that sodium nitrite regulates IL-1β in vivo. Future in vitro and in vivo studies using sodium nitrite exposure models are required to establish causality and clarify whether these candidate genes are directly affected by sodium nitrite or reflect downstream pathological processes in AS.

[This modification is located on page 23, lines 385-394]

**Comment 6: The reviewer requests that the SVM-RFE settings used for Fig 3 be reported in detail, including the kernel function (e.g., linear, RBF) and specific parameter configurations. In addition, the reviewer asks that performance metrics of the final selected model (e.g., Accuracy, Sensitivity, and Specificity) be provided.**

R:Thank you for your valuable suggestion. In response, we have revised the methodology section to clearly report the SVM-RFE configuration used in Figure 3. SVM-RFE is implemented using the e1071 package, and in the recursive feature elimination process, a linear kernel SVM is used to calculate feature weights (kernel="linear", type="C-classification"). The main parameters are set to cost (C)=10 and cache size=500. Because the expression matrix is standardized before modeling, internal scaling in SVM fitting is disabled (scale=False). Using conventional SVM-RFE (k=1), when the remaining number of features exceeds 5000, the features are halved in each iteration (halve. aovee=5000); Otherwise, the features will be sequentially deleted.In addition, to provide a more comprehensive evaluation of model performance beyond AUC, we have added other classification metrics for the final selected feature set in the results section, including accuracy, sensitivity, and specificity, which are derived from the same internal validation procedures described in the method. These indicators are now reported together with ROC analysis to improve the transparency and reproducibility of machine learning results.

**The revised content is as follows:** For SVM-RFE, feature ranking was implemented using the e1071 package (svm function) with a linear kernel (kernel = "linear", type = "C-classification"). The main parameters were set as cost (C) = 10 and cachesize = 500.Prior to SVM-RFE, the expression matrix was standardized; therefore, internal scaling in SVM fitting was disabled (scale = FALSE). Regular SVM-RFE was applied (k = 1), and when the number of remaining features exceeded 5000, features were removed by halving at each iteration (halve.above = 5000); otherwise, features were eliminated sequentially.

[This modification is located on page 5-6, lines 112-117]

In this external cohort, IL-1β showed the best-performing and most robust diagnostic discrimination, achieving an AUC of 0.822 (95% CI: 0.7372–0.9069, DeLong). Using the Youden index, the optimal cutoff (7.058918) yielded a sensitivity of 70.4% and a specificity of 84.8%.

[This modification is located on page 12, lines 230-233]

In this external cohort, IL-1β showed the best-performing and most robust diagnostic discrimination, achieving an AUC of 0.822 (95% CI: 0.7372–0.9069, DeLong). Using the Youden index, the optimal cutoff (7.058918) yielded a sensitivity of 70.4% and a specificity of 84.8%.

[This modification is located on page 12, lines 230-233]

**Comment 7: In molecular docking, results such as -6.9 kcal/mol (IL-1β) using AutoDock Vina have been reported.However, as this binding affinity is difficult to objectively consider strong, it would be advisable to present docking results with a Positive Control (an existing known IL-1β inhibitor or binding ligand) alongside these findings to demonstrate sodium nitrite's relative binding affinity to readers. Furthermore, regarding the description in the Results section, it is considered more appropriate in scientific literature to temper the expression ‘strong binding affinities’ to something like ‘moderate to strong’ or to present the values relative to the control group.**

R: Thank you for providing this helpful suggestion. We agree that the docking score in the report (such as IL-1 β being -6.9 kcal/mol) should be interpreted with caution, as overly strong wording may be misleading. Therefore, we have revised the results section by changing the description from "strong binding affinity" to "moderate (to strong) predicted binding affinity" and clarifying the use of docking values for relative comparison. The revised manuscript has updated the relevant text. At the same time, CANTOS clinical trial shows that kanamycin, an antibiotic targeting IL-1 β, can significantly reduce the recurrence of cardiovascular events, which further emphasizes the clinical relevance of IL-1 β in atherosclerosis. Therefore, based on these research findings, we believe that further validation of the binding affinity and mechanism of sodium nitrite to IL-1 β is of great significance.

**The revised content is as follows:** Sodium nitrite showed moderate to strong binding affinities with IL-1β, PTK2, IL6, and NOS3, with binding energies of -6.9, -6.7, -6.3, and -6.8 kcal/mol, respectively.

[This modification is located on page 15-16, lines 263-164]

Importantly, the CANTOS trial showed that targeting IL-1β with kanamycin can significantly reduce recurrent cardiovascular events, emphasizing its clinical relevance in atherosclerosis.

[This modification is located on page 21-22, lines 361-362]

**Comment 8: In Fig 8A, the protein backbone RMSD shows an initial increase followed by stabilization, whereas the Ligand RMSD (Fig 8B) exhibits greater variability.It would be beneficial to visually supplement this explanation using trajectory snapshots to clarify whether the ligand is stable within the binding pocket or exhibits tumbling motion within it.**

R: Thank you for providing this constructive suggestion. In the revised manuscript, we have improved the description of RMSD results to clearly distinguish between the initial equilibrium stage of the protein skeleton and the subsequent stable stage, while appropriately explaining the higher variability observed in ligand RMSD. In addition, the results section and legend have been revised to emphasize that ligand RMSD fluctuations reflect limited positional adjustments within the binding pocket, rather than global flipping or dissociation, which is supported by continuous hydrogen bonding/contact analysis. These revisions have improved the clarity and accuracy of MD trajectory interpretation.

**The revised content is as follows:** RMSD analysis showed an initial increase during equilibration followed by a plateau, indicating conformational adjustment and subsequent stabilization of the protein backbone. Overall, the backbone RMSD indicates stabilization after the initial equilibration phase (Fig. 9A). The RMSD profile of sodium nitrite indicated an initial exploratory phase within the binding pocket, followed by reduced fluctuations and a more confined conformational sampling,consistent with a confined binding mode (Fig. 9B). Root Mean Square Fluctuation (RMSF) analysis showed greater flexibility in loop regions and termini, whereas residues surrounding the binding pocket exhibited lower fluctuations around the binding region, supporting a relatively stable interaction pattern (Fig.9C). The Radius of Gyration (Rg) remained largely stable with only minor variations, indicating that IL-1β preserved its overall compactness and structural integrity during the simulation (Fig.9D). Solvent Accessible Surface Area (SASA) analysis displayed limited fluctuations, suggesting no drastic changes in protein folding or hydrophobic exposure, further supporting structural stability (Fig 9E). Finally, hydrogen-bond/contact analysis indicated persistent interactions between sodium nitrite and IL-1β, suggesting that the ligand remains associated with the pocket despite positional fluctuations(Fig. 9F)

[This modification is located on page 17, lines 272-288]

**Comment 9: This manuscript utilizes ssGSEA to confirm increased M0 macrophages, consistent with the pathology of AS.However, interpreting the results as ‘sodium nitrite induces immune infiltration’ is excessive.The current findings merely support that M0 levels are elevated in AS tissue and correlate with key genes.As this is not sodium nitrite exposure data, a more appropriate explanation would be along the lines of: ‘The target genes of sodium nitrite are closely associated with changes in the immune microenvironment of AS, suggesting a potential role in mediating toxicity.’**

R: We appreciate your important suggestion. We unanimously agree that our ssGSEA analysis was conducted in the AS cohort without information on sodium nitrite exposure; Therefore, causal wording such as' sodium nitrite induces immune infiltration 'is inappropriate. We have revised the results/discussion to describe the findings as an association: M0 macrophages are elevated in AS tissue and associated with key genes, indicating that target genes related to sodium nitrite may be associated with changes in the immune microenvironment of AS, rather than directly inducing immune infiltration.

**The revised content is as follows:** suggesting that sodium nitrite–related targets are associated with immune microenvironment changes in AS.

[This modification is located on page1-2, lines 23-24]

These findings suggest that sodium nitrite–related target genes are closely associated with immune microenvironment remodeling in AS, implying a potential role in mediating toxicity-related inflammatory pathways; however, causality requires validation in sodium nitrite exposure models.

[This modification is located on page 25, lines 441-444]

**Comment 10: While the Introduction section effectively outlines the risks of sodium nitrite, it would be beneficial to provide a more detailed explanation of why previous studies failed to elucidate the molecular mechanism. Furthermore, the statement ‘Sodium nitrite is generally considered protective...’ mentions the positive effects of low doses.To clearly delineate this from the study's focus on “toxicity”, it would be advisable to cite specific numerical values from prior research regarding the concentrations or conditions under which toxicity manifests.**

R: Thank you sincerely for your insightful feedback. We provided a more detailed explanation of the low-dose protective effects and high-dose toxicity of sodium nitrite in the introduction section, including the low-dose concentration range and high-dose toxicity related concentrations. Meanwhile, we cited relevant studies to clarify the dosage at which toxicity occurs under specific conditions. We believe that the reason why previous studies have not elucidated the molecular mechanism may be due to narrow research scope, incomplete experimental models, and a lack of interdisciplinary collaboration, resulting in a failure to fully reveal its mechanism. These supplements should help address the reviewers' concerns about dose and condition specificity.

**The revised content is as follows:** Clinically, sodium nitrite has mainly been used as an antidote under acute, closely monitored conditions, and its hemodynamic effects have been evaluated in controlled short-term infusion studies.In the human body, the toxicity of sodium nitrite is often associated with high-dose exposure, especially in suicidal sodium nitrite ingestion, where the nitrite concentration in the blood may reach 4.32 mM and high concentrations can cause the formation of high levels of MetHb, leading to hypoxia and multi organ damage.In experimental models and early-phase human studies, nitrite (including inhaled sodium nitrite formulations) has been evaluated as a nitric oxide donor capable of acutely lowering pulmonary pressures in pulmonary hypertension and as a cytoprotective agent in ischemia-reperfusion settings.

[This modification is located on page 2-3, lines 38-47]

Sodium nitrite exerts a dose-dependent dual effect on vascular health. In experimental settings, sodium nitrite may show a biphasic profile. In experimental settings, sodium nitrite may show a biphasic profile, with low doses (1-20 mM) acting through the NO pathway to promote vasodilation, reduce blood pressure, and prevent LDL oxidation. These effects help mitigate atherosclerosis progression. However, higher doses lead to the production of RNS, which induce oxidative stress and inflammation, accelerating atherosclerosis. The exact dose-response relationship and time-dependent effects require further investigation.

[This modification is located on page 23-24, lines 406-412]

**Comment 11: In the Discussion section, it would be beneficial to discuss the causal relationship between sodium nitrite→IL-1β binding→inflammatory response→AS induction in greater depth and detail, focusing on the AOP framework introduced by the authors and comparing it with prior literature.**

R: Thank you for your insightful feedback on the causal relationship between sodium nitrite, IL-1 β binding, inflammatory response, and AS induction. We have expanded this analysis in the revised discussion section, incorporating the Adverse Outcome Pathway (AOP) framework to clarify how exposure to sodium nitrite triggers inflammatory responses and accelerates AS progression through IL-1 β. We propose sodium nitrite as a molecular initiating event (MIE) that interacts with IL-1 β, potentially amplifying the inflammatory cascade associated with IL-1 β, promoting endothelial dysfunction and plaque instability. In addition, compared with previous studies, especially the findings of the CANTOS assay, we further support the central role of IL-1 β in AS and verify that sodium nitrite may accelerate the progression of AS by enhancing IL-1 β signaling. Through this extension, we aim to clarify the role of sodium nitrite in the pathogenesis of AS more clearly and emphasize the need for future in vitro and in vivo studies to validate this causal chain and potential therapeutic interventions of IL-1 β.

**The revised content is as follows:** Within the AOP framework proposed here, sodium nitrite exposure can be conceptually linked to AS through a stepwise causal cascade. As a putative molecular initiating event (MIE), our docking and MD results suggest a plausible interaction between sodium nitrite and IL-1β, which may affect IL-1β availability or downstream signaling potential, although the functional directionality requires biochemical and cellular validation. A subsequent key event (KE) is the amplification of vascular inflammatory signaling, characterized by elevated IL-1β–centered cytokine networks (e.g., TNF/IL-6) and activation of downstream pathways such as IL-17 and AGE–RAGE. These inflammatory KEs are expected to promote endothelial activation and chemokine-driven recruitment of monocytes/macrophages, consistent with our immune infiltration results showing increased macrophage-related signatures in AS plaques. Sustained immune cell infiltration and inflammatory signaling then represent later KEs that facilitate lipid dysregulation, foam cell formation, and plaque growth/instability, culminating in the adverse outcome (AO) of AS progression. Notably, prior studies have established IL-1β as a causal mediator in atherogenesis and plaque destabilization, and our findings extend this literature by proposing sodium nitrite as an upstream stressor that may converge on the IL-1β–inflammation axis in an AOP-consistent manner.

[This modification is located on page 26, lines 447-462]

**Comment 12:Throughout the manuscript, ‘Sodium nitrite’ and ‘sodium nitrite’ are used interchangeably.It is standard practice to use lowercase letters for chemical names unless they are at the beginning of a sentence.**

R: Thank you for your important suggestion. Regarding your point that the capitalization of 'Sodium nitrate/medium nitrate' in the entire manuscript is inconsistent. We agree that chemical names should be in lowercase unless they appear at the beginning of a sentence. Therefore, we standardized the term to 'sodium nitrate' throughout the manuscript and corrected the use of 'sodium nitrate' in the middle of sentences. This revision has been consistently applied in the abstract, methods (including section titles), results (including chart titles), and discussion. Additionally, according to formatting requirements, capitalized terms are only retained when they appear at the beginning of a sentence or in the title or keywords.

**Comment 13:This study relies entirely on computer simulations and public database analysis; biological validation through in vitro or in vivo experiments has not been performed.Experimental data verifying changes in mRNA or protein expression of key targets derived from sodium nitrite-treated cell models should be added.If this is not feasible, the limitations of the paper should be clearly stated, and the tone of the title or conclusion should be moderated to ‘Prediction’ or ‘In silico insights’.**

R: Thank you to the reviewer for their important suggestions on biological validation. We acknowledge that this study is based on computer simulations and public transcriptome datasets, and does not provide in vitro or in vivo evidence of changes in mRNA/protein expression after exposure to sodium nitrite. To address this limitation, we have strengthened the manuscript: firstly, it is explicitly stated in the discussion that external GEO validation supports IL-1 β as an AS marker, but does not prove that sodium nitrite regulates IL-1 β in vivo. We emphasize that the hypothesis of sodium nitrite → IL-1 β axis is still a hypothesis and needs experimental confirmation. Secondly, we have added explanations on the lack of cell and animal validation in the section on limitations and future prospects, and proposed specific experimental directions such as time series exposure experiments and animal tracking studies. Finally, due to the lack of wet laboratory validation, we adopted a "predictive/computer insights" tone in the abstract and conclusion to avoid exaggerating causal relationships.

**The revised content is as follows:** Collectively, this study proposed an adverse outcome pathway (AOP) framework linking sodium nitrite exposure to atherosclerosis, providing hypothesis-generating mechanistic insights for future experimental validation.

[This modification is located on page 2, lines 26-28]

This study systematically investigated, using computational approaches, potential mechanisms by which sodium nitrite may contribute to atherosclerosis. By integrating network toxicology, machine learning, molecular docking, and molecular dynamics simulations, we identified IL-1β, IL6, PTK2, and NOS3 as candidate targets, with stable in silico binding of sodium nitrite to IL-1β. The immune-infiltration patterns and the proposed AOP framework provide hypothesis-generating mechanistic insights that warrant validation in sodium nitrite exposure models.Collectively, our findings provide in silico evidence and a hypothesis-generating framework for assessing the potential cardiovascular risk of sodium nitrite exposure, highlighting the need for future experimental validation, especially under long-term low-dose exposure scenarios.

[This modification is located on page 27-28, lines 492-500]

We are deeply grateful for your guidance, which has greatly enhanced the quality of our manuscript. Should you have any additional comments or suggestions, please feel free to let us know.

Thank you once again for your valuable support.

Best regards,

Haobo Yang

**Reviewer#2**

**Dear Professor,**

**Comment 1: Sodium nitrite is used for cyanide poisoning, and can be used for patients with cardiovascular and arteriosclerosis, but the dosage should be reduced and the injection rate should be slowed down. However, the results of this study showed that sodium nitrite could promote atherosclerosis, which is inconsistent with the previous view, please explain the reason.**

R: Thank you for your important comment. We agree that sodium nitrite has known applications in clinical practice, such as as an antidote for cyanide poisoning, and may exert vascular protective effects under strictly controlled conditions. The difference from our research results mainly reflects the different exposure backgrounds, including dosage, route, and duration. In clinical practice, acute administration of sodium nitrite mainly exerts its effects through NO mediated vasodilation, and the dosage and infusion rate are strictly controlled. In our study, we focused on chronic exposure and high local nitrite load, which may lead to increased production of reactive nitrogen species, endothelial stress, and inflammatory response. In the AOP framework, we propose that sodium nitrite can amplify the inflammatory reaction centered on IL-1 β through interaction with IL-1 β, and then promote the progression of atherosclerosis. Although our computational simulations support this hypothesis, the causal relationship between sodium nitrite and IL-1 β still needs to be further validated through cellular and in vivo experiments. We have clarified the difference between therapeutic acute use and chronic exposure in the discussion and further improved this point.

**The revised content is as follows:** Clinically, sodium nitrite has mainly been used as an antidote under acute, closely monitored conditions, and its hemodynamic effects have been evaluated in controlled short-term infusion studies.In the human body, the toxicity of sodium nitrite is often associated with high-dose exposure, especially in suicidal sodium nitrite ingestion, where the nitrite concentration in the blood may reach 4.32 mM and high concentrations can cause the formation of high levels of MetHb, leading to hypoxia and multi organ damage.In experimental models and early-phase human studies, nitrite (including inhaled sodium nitrite formulations) has been evaluated as a nitric oxide donor capable of acutely lowering pulmonary pressures in pulmonary hypertension and as a cytoprotective agent in ischemia–reperfusion settings.

[This modification is located on page 2-3, lines 38-47]

**Comment 2: The relationship between sodium nitrite and atherosclerosis is complex, whether it ultimately plays a protective role or risk, whether there is epidemiological survey analysis or clinical verification, please supplement the relevant content.**

R: We appreciate your comment on the complexity of the relationship between sodium nitrite and cardiovascular health. In response, we acknowledge that the dual effects of sodium nitrite depend on the exposure environment, namely diet and acute medical use, source, and dosage. We have made appropriate revisions to the manuscript to better address this complexity. Specifically, we have expanded the discussion section to provide a clearer explanation of the relationship between consuming nitrite/nitrate from vegetables and reducing the risk of cardiovascular disease, as well as the relationship between nitrite in processed meat and the increased risk caused by the formation of harmful nitrosamines. In addition, we emphasize the need to consider exposure dose, duration, and source when determining the potential protective or harmful effects of sodium nitrite. We hope that the revisions we have made ensure that the manuscript reflects more clearly the complexity of the cardiovascular effects of sodium nitrite as suggested by the reviewers.

**The revised content is as follows:** Given the complexity of the effects of sodium nitrite, several epidemiological studies have explored the potential role of dietary nitrite/nitrate in cardiovascular health. A large-scale cohort study in 2025 found that excessive intake of plant nitrates is associated with a reduced risk of cardiovascular disease and mortality, possibly due to the beneficial effects of NO production on vascular function and health. However, the intake of nitrites from processed meats-where levels are much higher-has been associated with an increased risk of cardiovascular diseases. The presence of high levels of nitrites in processed foods is often linked to the formation of harmful nitrosamines under acidic conditions in the stomach. These compounds can lead to chronic inflammation and endothelial dysfunction, which contribute to the development of atherosclerosis.

[This modification is located on page 20-21, lines 331-340]

**Comment 3: Univariate and multivariate Cox regression analysis were used to study the relationship between survival time and potential risk factors for the disease. It is suggested that univariate and Cox regression analysis should be supplemented to verify the relationship between core genes, age, gender, race and other factors and the survival time of patients, and to clarify whether it is independent prognostic factor.**

R: Thank you for your valuable feedback on using univariate and multivariate Cox regression analysis to study the relationship between core genes, age, gender, race, and patient survival time. When reviewing the GSE28899 dataset, we found that it mainly consists of gene expression data and does not include detailed clinical variables such as survival time, age, gender, and race. These missing clinical variables prevent us from conducting Cox regression analysis to evaluate the relationship between these factors and survival time. We acknowledge this limitation in the limitations section of the manuscript. In future research, we plan to use a dataset that includes gene expression data and comprehensive clinical information to conduct a more thorough analysis of prognostic factors. Thank you for your understanding.

**Comment 4: Sodium nitrite showed strong binding affinities with IL-1β, PTK2, IL6, and NOS3, with binding energies of –6.9, –6.7, –6.3, and –6.8 kcal/mol, respectively. The binding energies of the four targets were not significantly, especially the binding energies of IL-1β and NOS3 were basically close, why only IL-1β was selected for the following experiments?**

R: Thank you for the insightful comments from the reviewer. Although the binding affinity between IL-1 β and NOS3 is similar, we understand the reviewer's concern about IL-1 β selection. In external validation using the GSE100927 dataset, IL-1 β showed the best diagnostic performance in core genes with an AUC of 0.822, significantly better than PTK2 (0.751), IL-6 (0.471), and NOS3 (0.618). Therefore, although the binding affinity of IL-1 β and NOS3 is similar, due to the key role of IL-1 β in diagnostic performance, atherosclerosis inflammation and its potential as a therapeutic target, it has been selected as the focus of further research. We have updated the discussion section to clarify this point and provide a theoretical basis for subsequent experimental research.

**The revised content is as follows:** To further address the potential risk of overfitting associated with single-cohort validation, we performed external validation using an independent GEO dataset (GSE100927). In this external cohort, IL-1β showed the best-performing and most robust diagnostic discrimination, achieving an AUC of 0.822 (95% CI: 0.7372–0.9069, DeLong). Using the Youden index, the optimal cutoff (7.058918) yielded a sensitivity of 70.4% and a specificity of 84.8%. In comparison, the AUCs of the other three genes in GSE100927 were PTK2 (0.751), IL6 (0.471), and NOS3 (0.618), indicating that IL-1β was the most consistently discriminative marker across datasets(Fig.6A-D).To evaluate the performance of the model, the accuracy, recall, and F1 score of the core objective were also calculated. The accuracy of IL-1β is 1, the recall rate is 0.875, and the F1 score is 0.933, showing excellent performance in distinguishing AS related biomarkers. These results emphasize the robustness of the model and support the diagnostic relevance of IL-1β in atherosclerosis.

[This modification is located on page 12, lines 229-239]

**Comment 5:This research screened out four core targets: IL-1β, PTK2, IL6 and NOS3. The characteristics of these four targets and research progress in the field of atherosclerosis should be elaborated and analyzed in the discussion section.**

R: Thank you for your valuable feedback. In response to your suggestion, we have provided a detailed introduction to the characteristics and research progress of four core targets (IL-1 β, PTK2, IL-6, and NOS3) in the discussion section. Each of these targets plays a crucial role in the pathogenesis of atherosclerosis by regulating vascular inflammation, immune cell recruitment, and plaque instability. We provided a detailed explanation of their biological functions and emphasized the latest research progress supporting their relevance in AS. These explanations and references are now included in the revised discussion section to clarify the role of each target in the pathogenesis of AS, consistent with recommendations for providing a more comprehensive analysis.

**The revised content is as follows:** In this study, we identified IL-1β, PTK2, IL-6, and NOS3 as core targets associated with AS progression. These four targets play important roles in regulating inflammation, endothelial dysfunction, and vascular remodeling. IL-1β is a key proinflammatory cytokine in the occurrence and spread of atherosclerosis plaque inflammation. Elevated levels of IL-1β are associated with increased endothelial dysfunction, immune cell infiltration, and plaque instability, all of which contribute to the progression of atherosclerosis. Importantly, the CANTOS trial showed that targeting IL-1β with kanamycin can significantly reduce recurrent cardiovascular events, emphasizing its clinical relevance in atherosclerosis. These findings are supported by extensive experimental evidence, positioning IL-1β as a key mediator of vascular inflammation and plaque instability, making it a promising therapeutic target for reducing AS related cardiovascular risk. PTK2 plays a crucial role in vascular remodeling, regulating cell adhesion, migration, and signal transduction, particularly in vascular smooth muscle cells and endothelial function. PTK2 (Focal Adhesion Kinase) activation can enhance vascular inflammation, promote immune cell recruitment, and facilitate plaque progression. This makes PTK2 an important target for stabilizing plaques and reducing the risk of plaque rupture. IL-6 is another pro-inflammatory cytokine involved in immune cell activation, acute phase response, and plaque formation. Elevated IL-6 levels are associated with vascular inflammation, plaque instability, and poor cardiovascular prognosis. It interacts with other inflammatory mediators such as TNF-α, amplifying the inflammatory response of the vascular system. Targeting IL-6 can help alleviate the inflammation that drives the progression of atherosclerosis. NOS3 is responsible for the production of NO. Nitric oxide is a molecule that regulates vasodilation, antithrombotic effects, and endothelial protection. The decrease in NOS3 activity leads to endothelial dysfunction, which is a hallmark of atherosclerosis. NOS3 dysfunction is associated with vascular stiffness, impaired vasodilation, and plaque instability, making it a potential therapeutic target for restoring endothelial function and slowing down the progression of atherosclerosis.

In conclusion, IL-1β, PTK2, IL-6 and NOS3 play different roles in vascular inflammation, immune cell recruitment and plaque instability, all of which are the core of atherosclerosis pathogenesis. Given their role in AS, these targets are valuable candidates for therapeutic interventions.

[This modification is located on page 21-22, lines 356-384]

**Comment 6:While LASSO regression was used, details about model robustness (e.g., external validation, overfitting control, data splitting strategy) are missing. Reporting performance on an independent test set and including more ML performance metrics (AUC, precision, recall, F1-score) is required.**

R: Thank you to the reviewer for their valuable feedback on the robustness and performance metrics of the model. In response to these comments, we have expanded the details of model validation, including: (i) external validation: validation using the independent GEO dataset GSE100927 to evaluate the model's generalization ability to new data. In the external validation, the AUC of IL-1 β is 0.822, which further supports its relevance as a diagnostic marker of atherosclerosis. (ii) Cross validation and data segmentation: Use 10 fold cross validation to ensure that model performance does not depend on specific subsets of data, select the best penalty parameter (λ), and reduce overfitting. (iii) Performance indicators: On the training dataset and external dataset (GSE100927), the AUC of IL-1 β is 0.822, the model accuracy is 1.0, the recall rate is 0.875, and the F1 score is 0.933, indicating that the model performs well in identifying true positives and minimizing false positives.

These indicators indicate that our LASSO regression model performs robustly on both the training and external datasets, and have been detailed in the revised manuscript to address the concerns of the reviewers and further validate the reliability of the model.

**The revised content is as follows:** To assess the robustness of the Lasso regression model, we included performance metrics such as AUC, precision, recall, and F1-score. In addition to the 10-fold cross-validation for model tuning and lambda selection, an independent external validation was performed using the GSE100927 dataset. The diagnostic performance was evaluated using ROC curves, and the AUC values were calculated for each of the core genes. Furthermore, classification performance was evaluated using precision, recall, F1-score, and the Youden index to assess sensitivity and specificity.

[This modification is located on page 6, lines 118-124]

To evaluate the performance of the model, the accuracy, recall, and F1 score of the core objective were also calculated. The accuracy of IL-1β is 1, the recall rate is 0.875, and the F1 score is 0.933, showing excellent performance in distinguishing AS related biomarkers. These results emphasize the robustness of the model and support the diagnostic relevance of IL-1β in atherosclerosis.

[This modification is located on page 12, lines 235-239]

We believe that these revisions have substantially improved the quality and clarity of our work. We respectfully submit the revised manuscript and the response document for your further evaluation.

Thank you very much for your consideration and continued support. We look forward to your positive response.

Warm regards,

Haobo Yang
