# Supplementary material for: Sodium nitrite promotes atherosclerosis via IL-1β: Network toxicology and machine learning insights
Source: PLoS One. 2026 May 5;21(5):e0347642. doi: 10.1371/journal.pone.0347642 (PMC13143078; doi:10.1371/journal.pone.0347642)
Supplement: Attachment — Submitted filename: Response_to_Reviewers_auresp_2.docx [file pone.0347642.s005.docx]

Journal：PLOS One

Manuscript Number:PONE-D-25-58627

Dear Editor of PLOS One,

Thank you very much for your letter and the reviewers' comments to improve the quality of the manuscript, based on which we have carefully revised the manuscript. The point-by-point responses to the comments are detailed in the following part. We hope these changes can make the manuscript more acceptable. There are two manuscript files, one is "Marked Revision", in which all the modifications are highlighted, and the other (Revised Manuscript) is the revised version ready for publication. If any further clarification and improvement are required, please do not hesitate to let us know. We look forward to hearing from you soon.

Note that the following color scheme is used in responding to reviewers' comments:

Black:reviewers' comments

**Blue:**our responses

***Blue:our revisions in the manuscript (italic)***

**Reviewer#1**

**Dear Professor,**

**Comment 1: In the response letter, the authors specified GeneCards' criteria as “Top 2000 genes... with a minimum correlation score threshold of 1.26.” However, the actual manuscript only states “the top 2000 genes based on relevance scores were selected,” omitting the specific score. Therefore, a brief supplementary description is needed in the manuscript.**

R: We sincerely thank the reviewer for this meticulous observation. As suggested, we have now explicitly included the specific correlation score threshold in the main text to ensure full clarity, and the following modifications are made in Section 2.2.

**The revised content is as follows:** For GeneCards, the top 2000 genes based on relevance scores were selected, using a minimum correlation score threshold of 1.26. In the case of STITCH, only interactions with a confidence score≥0.7 were considered.

[This modification is located on page 4-5, lines 91‒93.]

**Comment 2: Regarding the MD Simulation, it seems necessary to add a snapshot figure showing the actual ligand movement.**

R: We sincerely thank the reviewer for this valuable suggestion. As recommended, we have added a snapshot figure to visually demonstrate the movement and stability of the sodium nitrite ligand within the IL-1β binding pocket during the molecular dynamics simulation.

**The revised content is as follows:** Representative snapshots of the sodium nitrite binding pose within the IL-1β active site at 0, 25, 50, and 75 nanoseconds (Fig 9G) visually confirm the stable binding mode inferred from the quantitative analyses, showing minimal positional drift and consistent orientation of the ligand throughout the simulation trajectory. The superimposed alignment of these snapshots demonstrates minimal positional drift of sodium nitrite within the IL-1β binding pocket, indicating a stable binding mode throughout the simulation.

[This modification is located on page 18, lines 291-296.]

**Comment 3: This concerns the suggestion to perform comparative docking simulations using known inhibitors to compare binding affinity. While the authors reinforced their explanation by citing prior literature, the actual comparative docking data does not appear to have been added.**

R：We sincerely thank the reviewers for this valuable suggestion. Comparative docking analysis with known inhibitors can more effectively illustrate the specificity of sodium nitrite binding to the target. For this reason, we have carried out additional molecular docking experiments according to your suggestions.We chose MLN4924 to dock with IL-1 β, and compared its results with those of sodium nitrite. As shown in the figure, the binding mode and binding energy of mln4924 and IL-1 β active pocket (-8.9 kcal/mol) are different from sodium nitrite (-6.9 kcal/mol).This supplementary experiment intuitively showed the interaction differences between different ligands and IL-1 β, provided more direct comparative data support for our conclusion, and further enhanced the persuasion of the study.

**The revised content is as follows:** For comparative purposes, we also docked MLN4924, a known inhibitor of the IL-1β signaling pathway, to IL-1β. The significantly stronger binding energy of MLN4924 (-8.9 kcal/mol) provides a reference, indicating that the binding affinity of sodium nitrite for IL-1β is moderate to strong.

[This modification is located on page 27, lines 484‒487.]

We believe that these revisions have substantially improved the quality and clarity of our work. We respectfully submit the revised manuscript and the response document for your further evaluation.

Thank you very much for your consideration and continued support. We look forward to your positive response.

Warm regards,

Haobo Yang

**Reviewer#2**

**Dear Professor,**

**Comment 1: This study established an adverse outcome pathway (AOP) framework linking sodium nitrite exposure to the development of atherosclerosis. These findings provide new mechanistic insights and offer a theoretical foundation for assessing nitrite-associated cardiovascular risks.**

R：We sincerely thank the reviewer for their positive and encouraging comments on our work. We are pleased that they found our study to provide new mechanistic insights and a valuable theoretical foundation. Their acknowledgment is a great encouragement to our team.

Thank you very much for your consideration and continued support.

Warm regards,

Haobo Yang
