# Supplementary material for: Sodium nitrite promotes atherosclerosis via IL-1β: Network toxicology and machine learning insights
Source: PLoS One. 2026 May 5;21(5):e0347642. doi: 10.1371/journal.pone.0347642 (PMC13143078; doi:10.1371/journal.pone.0347642)
Supplement: Attachment — Submitted filename: Reviewer Comments_PONE-D-25-58627R2.docx [file pone.0347642.s004.docx]

**Title: Sodium nitrite promotes atherosclerosis via IL-1β: Network toxicology and machine learning insights. Manuscript Number: PONE-D-25-58627R2**

**Major Comments**

1. The manuscript still refers to the “top 2000 genes,” an issue previously raised by the reviewer and not addressed by the authors (Line 91).
2. The authors used AMBER for molecular dynamics simulations; why was CHARMM not used.
3. The authors state that a ‘de-identified public dataset’ was used; however, the reason for de-identification should be clearly explained.
4. The machine learning methodology is unclear, particularly regarding the target size; if only 24 targets were used, the dataset may be too small for reliable model training.
5. The hyperparameters used in the machine learning models are not reported and must be explicitly stated.
6. Although SVM & LASSO is mentioned, it is unclear whether other classifiers were tested; a comparative table reporting accuracy, RMSE, and correlation for all classifiers is needed to justify the choice of SVM.
7. The features used in the machine learning model and their total number are not clearly described, and any feature selection method applied should be specified.
8. Details of the machine learning validation strategy are missing, including the validation dataset and the training–testing split ratio.
9. The immune infiltration correlation analysis requires further explanation, particularly how the results demonstrate a significant increase in M0 macrophage infiltration in AS tissues compared to normal tissues.
10. A 2D interaction diagram for the molecular docking results should be provided, as conclusions based solely on docking scores are insufficient.
11. Figure 9 shows large RMSD fluctuations at multiple intervals, suggesting unstable docking; therefore, the molecular dynamics simulations should be reassessed or repeated.

**Minor Comments**

1. The Results section is brief and would benefit from more detailed explanation and interpretation.
2. Molecular dynamics plots should use nanoseconds (ns) as the time unit for consistency and clarity.
3. The peak observed in Figure 10B is unclear and requires a better viewing angle or improved representation.
