# Supplementary material for: Sodium nitrite promotes atherosclerosis via IL-1β: Network toxicology and machine learning insights
Source: PLoS One. 2026 May 5;21(5):e0347642. doi: 10.1371/journal.pone.0347642 (PMC13143078; doi:10.1371/journal.pone.0347642)
Supplement: Attachment — Submitted filename: Response_to_Reviewers_auresp_3.docx [file pone.0347642.s006.docx]

Journal：PLOS One

Manuscript Number:PONE-D-25-58627

Dear Editor of PLOS One,

Thank you very much for your letter and the reviewers' comments to improve the quality of the manuscript, based on which we have carefully revised the manuscript. The point-by-point responses to the comments are detailed in the following part. We hope these changes can make the manuscript more acceptable. There are two manuscript files, one is "Marked Revision", in which all the modifications are highlighted, and the other (Revised Manuscript) is the revised version ready for publication. If any further clarification and improvement are required, please do not hesitate to let us know. We look forward to hearing from you soon.

Note that the following color scheme is used in responding to reviewers' comments:

Black:reviewers' comments

**Blue:**our responses

***Blue:our revisions in the manuscript (italic)***

**Reviewer#2**

R：We sincerely thank the reviewer for their positive and encouraging comments on our work. We are pleased that they found our study to provide new mechanistic insights and a valuable theoretical foundation. Their acknowledgment is a great encouragement to our team.

Thank you very much for your consideration and continued support.

Warm regards,

Haobo Yang

**Reviewer#3**

**Dear Professor,**

**Comment 1: The manuscript still refers to the “top 2000 genes,” an issue previously raised by the reviewer and not addressed by the authors (Line 91).**

R: We sincerely thank the reviewer for raising this important point. As recommended, we have revised the manuscript to clarify the rationale for selecting the "top 2000 genes." Specifically, in lines 91-96 (Section 2.2), we now explicitly state that the top 2000 genes were identified from GeneCards based on a relevance score threshold of ≥1.26, which was applied to ensure the inclusion of genes with substantial association evidence while maintaining analytical tractability for downstream network and machine learning analyses. This revision provides a more transparent justification for the gene selection criteria, addressing the reviewer's concern.

**The revised content is as follows:** To balance comprehensiveness with analytical manageability, genes retrieved from GeneCards were ranked by their relevance scores. A threshold of relevance score ≥ 1.26 was applied to ensure the inclusion of genes with substantial association evidence. This threshold yielded the top 2000 ranked genes, which were selected for subsequent analysis. This approach was designed to capture a robust set of high-confidence candidate genes while maintaining a tractable dataset size for downstream network and machine learning analyses.

[This modification is located on page 5, lines 91-96.]

**Comment 2: The authors used AMBER for molecular dynamics simulations; why was CHARMM not used.**

R: We sincerely thank the reviewer for this careful observation, which allowed us to correct an oversight in the manuscript. The mention of the AMBER force field was a residual error from the early drafting phase. In our actual work, both the AMBER and CHARMM force fields were initially evaluated for the system. The CHARMM36 force field was ultimately selected for all production simulations as it provided better compatibility with the modeled ligand parameters and the TIP3P water model in our specific setup, leading to more stable simulation trajectories.

**The revised content is as follows:** The CHARMM36 force field was used to model biomolecular interactions, the system was solvated with the TIP3P water model, and Na⁺ ions were added to neutralize the overall charge.

[This modification is located on page 9, lines 190.]

**Comment 3: The authors state that a ‘de-identified public dataset’ was used; however, the reason for de-identification should be clearly explained.**

R：We sincerely thank the reviewer for this important comment regarding data ethics. As suggested, we have expanded the explanation in the manuscript to clarify the rationale for using a de-identified public dataset.

**The revised content is as follows:** The gene expression dataset (GSE28829) analyzed in this study was obtained from the Gene Expression Om nibus (GEO) public database on October 1, 2025. The source data had been fully de-identified prior to public deposition to protect participant privacy and confidentiality, in accordance with standard ethical requirements for sharing human genomic data. Consequently, the authors had no access to any information that could potentially identify individual participants throughout this research. The use of such pre-existing, anonymized public data for secondary analysis is in accordance with common institutional policies that allow for the waiver of ethical review and individual informed consent, as it involves no interaction with identifiable human subjects.

[This modification is located on page 9-10, lines 202‒210.]

**Comment 4: The machine learning methodology is unclear, particularly regarding the target size; if only 24 targets were used, the dataset may be too small for reliable model training.**

R：We sincerely thank the reviewer for this insightful comment regarding the machine learning methodology. We have revised the manuscript to provide a clearer explanation of our approach and the rationale behind the feature set size.

**The revised content is as follows:** The purpose of this machine learning (ML) step was to perform feature prioritization and refinement within a focused set of candidate targets. Specifically, the 24 overlapping sodium nitrite-AS candidate targets constituting the feature matrix were derived from the preceding network toxicology and database mining analyses (Sections 2.1–2.3), representing genes with prior biological plausibility for involvement in the mechanism of interest. Therefore, the application of LASSO and SVM-RFE was not intended for high-dimensional biomarker discovery from a full transcriptome, but rather to identify the most critical core drivers from this pre-filtered, biologically relevant candidate list. This approach of applying feature selection to a biologically curated gene set is a common strategy to enhance model interpretability and mitigate the risk of overfitting that can occur with high-dimensional data. The feature matrix for machine learning consisted of the expression values of 24 candidate genes in the GSE28829 dataset (n=29; AS=16, control=13). These 24 genes represent the overlapping targets between sodium nitrite-related genes and AS-associated genes, as identified through network toxicology analysis. Thus, the input features were pre-filtered based on biological plausibility, with a total of 24 features included in the initial model.

[This modification is located on page 5-6, lines 107‒121.]

**Comment 5: The hyperparameters used in the machine learning models are not reported and must be explicitly stated.**

R：We sincerely thank the reviewer for this constructive comment. In the original manuscript, the hyperparameters for SVM-RFE were provided, but the details for LASSO regression were not fully explicit. As recommended, we have now revised the manuscript to explicitly list all key hyperparameters for both models, with the LASSO regression parameters being the primary addition.

**The revised content is as follows:** For LASSO regression, the analysis was performed using the glmnetR package. The key parameters were set as follows: family = "binomial"for binary classification, alpha = 1to apply the L1 (LASSO) penalty, and standardize = TRUEto standardize features before model fitting. The optimal regularization parameter (λ) was determined via 10-fold cross-validation (nfolds = 10), with the specific λ value that minimized the cross-validation deviance (lambda.min) selected for the final model. A fixed random seed (e.g., set.seed = 123) was employed in the cross-validation procedures to ensure reproducibility.

[This modification is located on page 6-7, lines 138‒144.]

**Comment 6: Although SVM & LASSO is mentioned, it is unclear whether other classifiers were tested; a comparative table reporting accuracy, RMSE, and correlation for all classifiers is needed to justify the choice of SVM.**

R：We sincerely thank the reviewer for this constructive suggestion. In direct response, we have supplemented the study with a new comparative performance analysis of the two implemented algorithms (LASSO and SVM-RFE), detailed in Supplementary Table S1, which includes key metrics from cross-validation and external validation. This provides empirical support for our methodological choice. Concurrently, we have revised the manuscript to better clarify that the primary aim of the machine learning step was feature prioritization from a biologically pre-filtered gene set, for which LASSO and SVM-RFE are established tools. We have also transparently acknowledged in the "Limitations" section that a broader comparison with other classifiers represents a valuable direction for future research.

**The revised content is as follows:** (5)Regarding the machine learning approach, this study employed a combination of LASSO regression and SVM-RFE. While these algorithms were selected based on their methodological strengths (LASSO for regularization and feature selection, SVM-RFE for robust feature ranking in linearly separable data), we acknowledge that systematic comparison with other classifiers—such as random forest, XGBoost, or neural networks—was not performed. This represents a limitation of the present study. Future research should incorporate a broader range of machine learning algorithms for comparative evaluation to further validate and optimize the feature selection pipeline.

[This modification is located on page 31-32, lines 566‒573.]

**Comment 7: The features used in the machine learning model and their total number are not clearly described, and any feature selection method applied should be specified.**

R：We sincerely thank the reviewer for this comment, which has helped us improve the clarity of our methodology section. In direct response, we have revised the manuscript to explicitly and concisely state the total number of features, their origin, and the feature selection methods applied.

**The revised content is as follows:** Two machine learning algorithms were employed to perform feature selection and identify core genes associated with sodium nitrite-induced AS: LASSO regression and SVM-RFE. LASSO regression integrates built-in feature selection with L1 regularization, shrinking the coefficients of less important features to zero and thereby selecting a subset of predictive genes. SVM-RFE is a wrapper-based feature selection method that iteratively removes the least important features based on SVM weight coefficients, identifying the optimal feature subset through cross-validation. Both methods were applied to the 24-feature matrix to further refine the gene set.Core target genes were defined as the intersection of genes identified by both algorithms, resulting in a final set of four features (IL-1β, IL6, PTK2, and NOS3) that were considered the most robust candidates for subsequent analyses.

[This modification is located on page 6, lines 121‒130.]

**Comment 8: Details of the machine learning validation strategy are missing, including the validation dataset and the training–testing split ratio.**

R：We thank the reviewer for this important comment. We have revised the Methods section (2.4 Screening of core target genes using machine learning algorithms) to provide complete details of the validation strategy, including the specific internal validation method and the external validation dataset.

**The revised content is as follows:** For internal validation, a 10-fold cross-validation strategy was employed. The GSE28829 dataset was randomly partitioned into 10 approximately equal-sized subsets. In each iteration, nine subsets were used for model training and the remaining subset for validation, with the process repeated ten times to ensure all samples were used for validation once. The average performance metrics across folds were reported. For external validation, the fully trained model (using all GSE28829 samples) was applied to an independent dataset, GSE100927. This independent evaluation assesses the generalizability of the identified core genes.

[This modification is located on page 7, lines 145‒151.]

**Comment 9: The immune infiltration correlation analysis requires further explanation, particularly how the results demonstrate a significant increase in M0 macrophage infiltration in AS tissues compared to normal tissues.**

R：We thank the reviewer for this comment. We have revised the manuscript to provide a clearer, step-by-step explanation of the immune infiltration analysis and the basis for the conclusion regarding M0 macrophages.

**The revised content is as follows:** To elucidate the immune microenvironment in AS, we employed single-sample Gene Set Enrichment Analysis (ssGSEA) to quantify the relative infiltration levels of 21 immune cell types in AS plaque tissues (n=27) compared to normal arterial tissues (n=14). The overall distribution of immune cells is visualized in Figure 7A. Statistical comparison using the Wilcoxon rank-sum test revealed significant differences in the infiltration of specific immune subsets between the two groups (Figure 7C). Crucially, we observed a statistically significant increase in the infiltration of non-polarized (M0) macrophages in AS tissues compared to normal controls (p < 0.001), suggesting their potential role in initiating and sustaining plaque inflammation. In contrast, the infiltration levels of neutrophils, M1 macrophages, M2 macrophages, regulatory T cells (Tregs), and resting NK cells were significantly higher in normal tissues (all p < 0.05). Furthermore, Spearman correlation analysis was performed to assess the interrelationships among different immune cell types (Figure 7B). Finally, to explore the mechanistic links between the core targets and the immune landscape, we evaluated the correlations between the expression of the four core genes (IL-1β, PTK2, IL6, NOS3) and the infiltration levels of these differentially abundant immune cells.

[This modification is located on page 17, lines 292‒306.]

**Comment 10: A 2D interaction diagram for the molecular docking results should be provided, as conclusions based solely on docking scores are insufficient.**

R：We sincerely thank the reviewer for this constructive suggestion. We fully agree that detailed intermolecular interaction diagrams are crucial for elucidating binding modes beyond docking scores alone. In response, we have made three key revisions throughout the manuscript: (1) In the Methods section, we described the generation of 2D interaction diagrams using LigPlot+; (2) In the Results section, we added a new paragraph detailing the specific 2D interaction patterns (e.g., hydrogen bonds, salt bridges) between sodium nitrite and the core targets (IL-1β, PTK2, IL6, NOS3), as well as with the positive control MLN4924; (3) In the Discussion, we incorporated the specific interactions revealed by the 2D diagram (such as the salt bridge with Arg A:4 in the IL-1β binding pocket) to provide structural rationale for the inference that sodium nitrite may directly target IL-1β. These revisions comprehensively present and deepen the analysis of the binding modes from methodology to interpretation, thereby strengthening the persuasiveness of the study.

**The revised content is as follows:** Following 3D visualization, 2D diagrams depicting the specific molecular interactions between sodium nitrite and the target proteins were generated using LigPlot+. These diagrams provide a schematic representation of the binding interactions for further analysis.

[This modification is located on page 8, lines 182‒184.]

**The revised content is as follows:** To elucidate the specific molecular binding mode, a 2D ligand protein interaction diagram was generated. Analysis shows that sodium nitrite mainly binds to the core target through hydrogen bonding, salt bridging, and hydrophobic interactions. Among them, the interaction with IL-1β (binding energy -6.9 kcal/mol) is the most significant, forming a stable salt bridge with the key residue Arg A: 4 and hydrogen bonding with Gln A: 48, which provides a structural explanation for its binding affinity. The 2D images of PTK2, IL6, and NOS3 also reveal similar specific interaction networks. In addition, the docking results of the positive control MLN4924 with IL-1β showed a broader interaction, verifying the reliability of the docking method, while highlighting the different and more focused binding characteristics of sodium nitrite. These structural details collectively reinforce the evidence that sodium nitrite can directly act on these core targets.

[This modification is located on page 19, lines 318‒328.]

**The revised content is as follows:** The 2D interaction diagram further clarified that this high-affinity binding is mediated by a stable salt bridge with the key residue Arg A:4 and a hydrogen bond with Gln A:48 within the IL-1β binding pocket, providing structural rationale for the predicted interaction.

[This modification is located on page 29, lines 526‒528.]

**Comment 11: Figure 9 shows large RMSD fluctuations at multiple intervals, suggesting unstable docking; therefore, the molecular dynamics simulations should be reassessed or repeated.**

R：We sincerely thank the reviewer for this careful observation. In response, we have revised the manuscript to provide a more detailed and integrated interpretation of the molecular dynamics simulation data, particularly regarding the RMSD profile and the overall assessment of stability.

**The revised content is as follows:** To further examine the stability of protein--ligand interactions, MD simulations were conducted for the IL-1β--sodium nitrite complex. Multiple independent metrics were analyzed to comprehensively assess the structural stability and binding persistence of the complex. Protein backbone RMSD analysis (Fig 9A) revealed a stable trajectory throughout the 100 ns simulation. Following an initial equilibration phase during the first 20 ns, the RMSD stabilized and remained within a narrow range (approximately 0.2–0.3 nm) with no major fluctuations, indicating that the protein maintained its structural integrity. Ligand RMSD analysis (Fig 9B) showed moderate fluctuations during the first 40 ns, which correspond to the ligand exploring its binding pose within the pocket—a common phenomenon in protein–ligand simulations. After this initial sampling period, the ligand RMSD stabilized and remained confined for the latter 60 ns, indicating the establishment of a stable binding mode. Critically, the radius of gyration (Rg) remained largely constant throughout the simulation (Fig 9D), demonstrating that the protein retained its compact, globular structure and did not undergo global unfolding. This is a key indicator that the observed backbone RMSD changes reflect localized conformational adjustments rather than overall destabilization. The solvent-accessible surface area (SASA) displayed only minor fluctuations (Fig 9E), further supporting the maintenance of structural integrity. Root mean square fluctuation (RMSF) analysis (Fig 9C) showed expected flexibility in loop regions and termini, while residues surrounding the binding pocket exhibited lower fluctuations, supporting a stable interaction interface. Most importantly, hydrogen bond analysis (Fig 9F) revealed persistent interactions between sodium nitrite and IL-1β throughout the simulation, confirming that the ligand remained associated with the binding pocket despite positional fluctuations[^[1]^]. Representative snapshots of the sodium nitrite binding pose at 0, 25, 50, and 75 ns (Fig 9G) visually confirm the stable binding mode inferred from the quantitative analyses, showing minimal positional drift and consistent orientation of the ligand throughout the trajectory. Collectively, these multiple lines of evidence—stable Rg, persistent hydrogen bonds, confined ligand RMSD after initial sampling, and localized RMSF patterns—demonstrate that the RMSD variations observed reflect expected dynamic conformational adjustments rather than unstable binding. The system reached a stable equilibrium, validating the overall stability of the sodium nitrite–IL-1β complex.

[This modification is located on page 20-22, lines 332‒359.]

**Comment 12: The Results section is brief and would benefit from more detailed explanation and interpretation.**

R：We sincerely thank the reviewers for their valuable suggestions. We have revised the Results section (Section 3) and included more detailed explanations, biological background, and explanatory statements.

**The revised content is as follows:** The identification of these four genes by both LASSO and SVM-RFE algorithms increases confidence in their relevance to sodium nitrite-induced AS. IL-1β and IL6 are well-established pro-inflammatory cytokines central to atherosclerosis, while PTK2 (focal adhesion kinase) plays a role in vascular remodeling and NOS3 regulates endothelial function through nitric oxide production. The convergence of machine learning results on these mechanistically plausible targets supports the validity of our feature selection approach.

[This modification is located on page 13, lines 247‒252.]

**The revised content is as follows:** For comparative purposes, we also docked MLN4924, a known inhibitor of the IL-1β signaling pathway, to IL-1β. The significantly stronger binding energy of MLN4924 (-8.9 kcal/mol) provides a reference, indicating that the binding affinity of sodium nitrite for IL-1β is moderate to strong. These binding energies, all below the –5.0 kcal/mol threshold for spontaneous binding, suggest that sodium nitrite has the structural potential to directly interact with all four core targets. The strongest affinity observed for IL-1β (–6.9 kcal/mol) is particularly noteworthy, as it aligns with the central role of IL-1β in our subsequent MD simulations and immune infiltration analyses. While docking scores alone cannot confirm biological activity, they provide a basis for hypothesizing direct molecular interactions that warrant experimental validation.

[This modification is located on page 19, lines 318‒327.]

**Comment 13: Molecular dynamics plots should use nanoseconds (ns) as the time unit for consistency and clarity.**

R：We thank the reviewer for this important correction. We have revised all molecular dynamics simulation plots (specifically Figure 9 and any other related figures) to consistently use nanoseconds (ns) as the time unit on the x-axis, replacing the previously used picoseconds (ps). The data has been rescaled accordingly (1 ns = 1000 ps).

[This modification is located on page 22, Fig 9.]

**Comment 14: The peak observed in Figure 10B is unclear and requires a better viewing angle or improved representation.**

R：We thank the reviewer for this helpful suggestion to improve the clarity of the figure. In direct response, we have revised Figure 10Bby adjusting and optimizing the viewing angle/rotation of the 3D representation to more clearly display the key structural feature (peak) in question.

**The revised content is as follows:** The presence of two distinct energy minima corresponding to unbound and bound states, separated by a moderate energy barrier (12.5–17.5 kJ/mol), indicates that sodium nitrite binding to IL-1β is both thermodynamically favorable and kinetically accessible. The deep, narrow energy well in the bound state region (~5 kJ/mol) further supports the high stability of the complex observed in the RMSD and hydrogen bond analyses. This energy landscape is characteristic of specific, biologically relevant protein–ligand interactions rather than non-specific, transient binding.

[This modification is located on page 23, lines 380‒386.Figure 10B]

We believe that these revisions have substantially improved the quality and clarity of our work. We respectfully submit the revised manuscript and the response document for your further evaluation.

Thank you very much for your consideration and continued support. We look forward to your positive response.

Warm regards,

Haobo Yang

**Reviewer#4**

**Dear Professor,**

**Comment 1: Please correct a factual error regarding the CANTOS trial: the manuscript currently states “targeting IL-1β with kanamycin” , which should be revised to the correct intervention (canakinumab).**

R：We sincerely thank the reviewer for identifying this critical error and for providing the precise correction. We have revised the manuscript accordingly.

**The revised content is as follows:** Importantly, the CANTOS trial showed that targeting IL-1β with canakinumab can significantly reduce recurrent cardiovascular events, emphasizing its clinical relevance in atherosclerosis.

[This modification is located on page 26, lines 441.]

Thank you very much for your consideration and continued support.

Warm regards,

Haobo Yang
